# Home care after intensive care unit-discharge: global differences

**DOI:** 10.62675/2965-2774.20250269

**Published:** 2025-05-07

**Authors:** Cassiano Teixeira, Regis Goulart Rosa

**Affiliations:** 1 Universidade Federal de Ciências da Saúde de Porto Alegre Department of Internal Medicine Porto Alegre RS Brazil Department of Internal Medicine, Universidade Federal de Ciências da Saúde de Porto Alegre - Porto Alegre (RS), Brazil.; 2 Universidade Federal do Rio Grande do Sul Hospital de Clínicas de Porto Alegre Intensive Care Unit Porto Alegre RS Brazil Intensive Care Unit, Hospital de Clínicas de Porto Alegre, Universidade Federal do Rio Grande do Sul - Porto Alegre (RS), Brazil.; 3 Universidade Federal de Ciências da Saúde de Porto Alegre Postgraduate Program in Rehabilitation Sciences Porto Alegre RS Brazil Postgraduate Program in Rehabilitation Sciences, Universidade Federal de Ciências da Saúde de Porto Alegre - Porto Alegre (RS), Brazil.; 4 Hospital Moinhos de Vento Department of Internal Medicine Porto Alegre RS Brazil Department of Internal Medicine, Hospital Moinhos de Vento - Porto Alegre (RS), Brazil.

**Keywords:** Homecare, Aftercare, Discharge, Hospitals, Quality of life, Delivery of health care, Social support, Health inequities, Home care services, Comprehensive health care, Intensive care units

## Abstract

Significant physical and neuropsychiatric challenges, diminished life quality, and heightened demand for healthcare services often mark the period following discharge from the intensive care unit and hospitalization. Most follow-up care for these patients relies on clinic-based models, necessitating visits to healthcare facilities for rehabilitation and continued monitoring. However, this approach can create barriers for the most severely affected individuals, potentially worsening health inequities. In contrast, home care offers a viable solution by providing essential social support and assistance to patients with varying healthcare needs, allowing them to preserve their independence within the familiar environment of their own homes and communities. This model presents a promising alternative to the conventional clinic-based post-intensive care unit recovery system. It is cost-effective and better aligned with the preferences of an increasing number of individuals who choose to stay at home rather than move to institutional settings for care.

## INTRODUCTION

### A global emerging issue

The impact on both the quantity^(1)^ and quality^(2-4)^ of life for patients who survive acute critical illnesses has become a significant concern for intensivists and health authorities across various nations.^(5,6)^ Traditionally, intensive care has been focused on minimizing immediate mortality risks; however, those who survive often face substantial risks of mortality over the medium and long term. Additionally, they experience a range of physical complications, cognitive impairments, mental health issues, and sexual dysfunction after leaving the intensive care unit (ICU).^(6-10)^ Beyond this, the recovery phase is typically characterized by frequent hospital readmissions and high demand for healthcare services, placing a considerable financial strain on healthcare systems.^(11-13)^

The trend toward shorter hospital stays, compounded by financial constraints, has limited hospital staff's ability, including nurses, to sufficiently prepare patients and their caregivers for the transition from the hospital to home and the ongoing care needs that follow. As a result, many individuals discharged from the ICU or hospital are left unprepared to manage their healthcare independently. They are often uncertain about how to proceed or whom to contact if their condition worsens.^(14)^ Similarly, caregivers frequently lack adequate training to oversee prescribed treatments and necessary lifestyle modifications. These patients may struggle to seek help when they encounter care-related issues or may fail to notice subtle health changes before they develop into more serious symptoms.^(15,16)^ Furthermore, these patients face a significantly higher risk of being readmitted to the hospital, with approximately 15% to 20% of Medicare patients being readmitted within 30 days of discharge, and this figure rises to 25% to 30% for patients with sepsis.^(17-19)^ Such patients are often called "hospital-dependent" individuals.^(20)^

Several factors contribute to this issue, such as insufficient discharge planning from the hospital or ICU, poor medication reconciliation, limited access to follow-up care (e.g., delays in primary care and specialist appointments), and confusion regarding post-discharge responsibilities of healthcare providers. This has become a recognized gap in the healthcare system, highlighting the failure to provide continuous, coordinated, and comprehensive care. In response, hospitals and healthcare organizations have implemented various strategies, including care coordinators, post-discharge pharmacists, transition coaches, and after-hours clinics. However, these interventions have not yet achieved the desired level of success.^(21-23)^

### Post-discharge care interventions

Despite a growing body of research on discharge care, healthcare providers face difficulties in determining the most appropriate interventions and timing following hospital discharge. These interventions, initiated by the hospital, involve engaging patients shortly after they leave the hospital through strategies like scheduled follow-up calls, home visits, and appointments at outpatient clinics. The educational component of these interventions typically covers a range of topics, including medication management, healthy eating, self-care for specific conditions, physical activity, and the importance of follow-up care.^(24)^ Various tools, including paper and digital resources, toolkits, and health tracking logs, support patients after discharge, helping them adhere to discharge plans and manage emerging health concerns. The primary objectives of these hospital-driven post-discharge interventions are to reduce disease burden, prevent readmissions, and ultimately improve the quality of life for both patients and their caregivers.^(25,26)^

While the severity of patients’ conditions following critical illness can complicate their ability to attend medical appointments, the predominant approach to post-ICU follow-up still revolves around clinic-based models. These models require patients to visit healthcare facilities for rehabilitation and follow-up services.^(27)^ Ironically, this structure may inadvertently prevent the most severely ill patients from receiving adequate care, thus exacerbating existing health disparities.^(28)^

Maintaining close follow-up with patients after discharge is crucial to ensuring their health and safety outside the hospital. This ongoing contact can help prevent further complications, ensure that patients receive timely medical attention, and enable the early identification of concerning health issues. Ideally, community-based or outpatient healthcare providers would have the necessary resources to maintain ongoing communication with patients through in-person visits, phone calls, or other means to address any health-related issues that arise post-discharge. However, in some instances, hospital clinicians may be better suited to facilitate or provide such contact, mainly if they were involved in the patient's care during their hospital stay or had access to medical records that could aid in post-discharge management. Despite previous difficulties in reimbursement under fee-for-service models, engaging hospital staff or existing home care teams to conduct post-discharge visits could prove more effective and efficient. Home visits provide an opportunity to assess the patient's living environment, identify potential obstacles to recovery, and address issues that may otherwise go unnoticed. Additionally, these visits can help strengthen the relationship between patients and healthcare providers, fostering better trust and communication during recovery.

### Home healthcare services

Home health care offers crucial social support and assistance to individuals with varying healthcare needs, helping them retain as much independence as possible within the comfort of their own homes and communities. This model has become an increasingly appealing option for policymakers due to its cost-effectiveness and alignment with the growing preference of many individuals to stay in their homes rather than move to residential care facilities.^(29)^ Home healthcare programs share common characteristics in countries such as Australia, Germany, and Canada. These programs are often publicly funded, comprehensive (offering post-acute, supportive, and end-of-life care within a single program), use need-based eligibility criteria instead of income-based criteria, and typically have a single entry point for service access.^(30)^

It is important to emphasize that home care is a specialized healthcare service requiring skilled professionals. Adequate home care involves technical expertise and strong interpersonal skills, as professionals must work closely with patients, families, and multidisciplinary teams. Home care providers need to be autonomous, responsible, and well-versed in healthcare's technical and scientific aspects. As a result, home care professionals must continually develop a wide range of skills and remain committed to further education to meet the demands of this multifaceted field.^(31)^

In the United States, Medicare home health care covers various services, including skilled nursing, physical therapy, occupational therapy, speech therapy, aide services, and medical social work, all delivered in the patient's home. To be eligible for Medicare's home health benefit, beneficiaries must require part-time or intermittent skilled care due to illness or injury and face significant difficulty leaving home. Unlike skilled nursing facility coverage, Medicare does not require a prior hospital stay to qualify for home health care. Additionally, home health services through Medicare do not have copayments or deductibles. In 2016, approximately 3.4 million Medicare beneficiaries received home care, and the program spent $18.1 billion on these services. Medicare's spending on home health care has more than doubled since 2001 and now constitutes about 4.6% of the total fee-for-service expenditure.^(32)^

The Federal Government's Home Support Program is the primary service for older individuals requiring home care in Australia. Around 5% of older Australians use basic home care services through this program, which supports individuals aged 65 years or older or Indigenous Australians aged 50 years or older who suffer from chronic illnesses, disabilities, or physical and cognitive decline.^(29)^ The program provides services to help maintain independent living, such as meal preparation, personal care, and domestic assistance. While the program addresses aging-related needs, home-based disability support in Australia follows different funding and administrative guidelines.^(29)^ Although precise statistics on the recipients of home care and their needs are sometimes lacking, there is a clear correlation between an aging population and an increase in chronic conditions like depression, which is often undiagnosed in elderly individuals (Table 1).

**Table 1:** Australian home care recipients^(29)^

− Frail-aged (i.e., over 65 years if non-indigenous, over 50 years if Indigenous Australian) access bulk of home care services
− Disability—e.g., intellectual, physical or psychiatric
− Any combination of frail aged, chronic illness, and disability
− Indigenous Australians

In Canada, the home care process begins with a referral, after which a case manager is assigned to assess the client and potential caregivers, coordinate the necessary services, authorize them, and conduct ongoing monitoring. Home care providers in Canada typically include personal support workers and nurses, whom public health services or private agencies may employ. Personal support workers assist with daily living activities, while nurses provide more specialized clinical care. The home care team may include occupational therapists, physiotherapists, pharmacists, nurse practitioners, social workers, dietitians, and physicians. In Canada, personal support workers are the primary providers of home care services, serving 50% to 69% of clients.^(33)^

In Germany, all residents are covered by mandatory health insurance, including home care services coverage. The process begins with a needs assessment conducted by a physician or specialized team, who recommends a care plan and may refer the patient to a home care agency.^(34)^ These agencies offer various services, from assistance with daily activities such as eating and bathing to more complex care, including medication management and support for chronic conditions. Specialized care, including palliative care, is also available. Funding is partially covered by health insurance, which may require patient copayments. For extended care, the *Pflegeversicherung* covers additional expenses. Care coordination and monitoring are managed by professionals who ensure the care plan is followed and adjusted as needed. The patient's progress is regularly reviewed to meet their needs. Patients have the right to quality care and the choice of care providers. The system also supports families in integrating home care into daily life, facilitating a smooth transition and ensuring appropriate support.^(34)^

In Brazil, a middle-income country, the "*Programa Melhor em Casa*" is a public home care initiative designed to achieve several objectives: (1) to promote the dehospitalization of stable patients, enabling them to continue their healthcare at home, particularly when the complexity of care required exceeds that which primary care can provide; (2) to prevent hospitalization of patients originating from primary or emergency care; and (3) to reduce hospital readmissions. The program caters to patients classified into care levels AD2 and AD3, as per the Ordinance of Home Care issued by the Brazilian Ministry of Health (Table 2). The program offers to individuals who face temporary or permanent difficulties leaving their homes to access healthcare facilities or to whom home care is deemed the most appropriate treatment option. Home care aims to provide patients with care that integrates into their family routine, minimizing unnecessary hospitalizations, reducing the risk of infections, and allowing patients to remain with their loved ones at home. In cases in which patients require frequent visits, specialized home care teams may accompany them. These teams typically consist of multidisciplinary professionals, including doctors, nurses, nursing technicians, physical therapists, and social workers. Additionally, support teams may comprise other professionals such as speech therapists, nutritionists, dentists, psychologists, occupational therapists, and pharmacists. Each team can typically manage approximately 60 patients concurrently. Home care teams are employed by states and municipalities and operate on a Monday-to-Friday schedule, working 12-hour shifts daily. Health care is also available on weekends and holidays through on-call services. Only cities and states with qualified teams registered in the National Register System and consistently reporting attendance information to the Primary Health Care Information System can receive funding from the Ministry.^(35)^ However, the outcomes of this program have not yet been published.

**Table 2:** Indications for "*Programa Melhor em Casa*" based on Brazilian home care classification criteria^(35)^

AD2*	AD3†
Demand for more complex procedures that can be performed at home, such as: − Complex dressings (levels 3 and 4) and abscess drainage, among others − Dependence on frequent monitoring of vital signs/ unstable conditions − Frequent and systematic need for less complex laboratory tests − Adaptation of the user and/or caregiver to the use of the tracheostomy device − User adaptation to the use of orthoses/prostheses − Adaptation of users to the use of probes and ostomies − Postoperative home follow-up, as indicated by the surgical team − Rehabilitation of people with permanent or transient disabilities who need frequent care until they are able to attend rehabilitation services − Use of an airway aspirator for bronchial hygiene − Need for permanent or transient nutritional attention − Frequent care in terminal patients/pain relief measures − Need for intravenous or subcutaneous medication	The existence of at least one of the situations accepted as inclusion criteria for care in AD2 modality and the need to use at least one of the following equipment/procedures: − Noninvasive ventilatory support (continuous positive airway pressure or bi-level positive airway pressure) − Peritoneal dialysis − Paracentesis − Use of total parenteral nutrition

* The AD2 classification refers to patients who require home care with a moderate level of complexity. These patients typically need assistance with Basic Daily Activities, such as eating, bathing, mobility, monitoring health conditions, and medication administration. Typically included care: assistance with daily activities, medication administration, monitoring of vital signs, and support with health-related tasks, but without the need for highly specialized or complex medical care.

† The AD3 classification is for patients who require home care with a higher level of complexity. These patients have more extensive needs and may require advanced or specialized medical care. Typically included care: in addition to assistance with daily activities and medication administration, AD3 patients may need more intensive care, such as respiratory support, complex wound care, monitoring of severe chronic conditions, and palliative care.

### Home healthcare outcomes

Interestingly, many patients and their families often prefer home health care as a viable option. Most home health care agencies encourage family members, loved ones, and patients to acquire basic skills needed to manage some aspects of the care, such as personal care, wound management, and administering intravenous medications. While home health care has the potential to reduce certain healthcare costs, particularly those related to hospitalization or long-term institutional care, it can also create personal burdens. Family members may experience emotional, social, physical, and financial strain. The effectiveness of home health care can be jeopardized if the patient's informal support network becomes overburdened, mainly due to disease progression, intensified treatment, or depletion of resources. Home health care does not always result in cost savings for patients with insurance. The expenses for family caregiving and out-of-pocket costs that are not reimbursed may lead to higher immediate costs than inpatient hospitalization. Furthermore, the quality of care is significantly influenced by the ability of healthcare providers to gather and address concerns from patients and their families, as well as through direct communication between physicians and family members.

A recent meta-analysis of 20 clinical trials examined the impact of home visits and follow-up phone calls, finding that these interventions were associated with a reduced risk of hospital readmissions.^(24)^ Specifically, the analysis highlighted that home visits and follow-up calls were among the most effective interventions. Patients who received two or more home visits had a lower likelihood of being readmitted (OR 0.6 [95%CI 0.4 - 0.7]) than those who did not receive home visits. In contrast, having just one home visit did not show a significant effect (OR 1.0 [95%CI 0.8 - 1.1]). Among patients who received two or more post-discharge home visits, the readmission rate was 24% (95%CI 16 - 34%), compared to 36% (95%CI 26 - 48%) for those without visits.^(24)^ Similarly, having two or more follow-up phone calls was associated with a significantly reduced risk of readmission (OR 0.7 [95%CI 0.6 - 0.8]). However, one phone call alone did not appear to have a meaningful effect on readmission rates (OR 0.9 [95%CI 0.7 - 1.1]). For patients who received two or more follow-up phone calls, the readmission rate was 23% (95%CI 15 - 35%), compared to 31% (95%CI 20 - 45%) for those who did not receive any calls.^(24)^ When adjustments were made for the length of follow-up, patient diagnoses, and discharge education exposure, the likelihood of readmission was significantly lower for patients who received both multiple home visits and follow-up phone calls (OR 0.5 [95%CI 0.4 - 0.7]). In conclusion, the results suggest frequent post-discharge home visits and follow-up phone calls are crucial in reducing readmission rates. Furthermore, patients who participated in discharge education interventions had a lower risk of readmission (OR 0.7 [95%CI 0.6 - 0.8]), with a readmission rate of 27% (95%CI 19 - 36%) among those exposed to the intervention, compared to 34% (95%CI 26 - 45%) among those who were not.^(24)^

### Barriers

Adequate home care following hospital discharge poses numerous challenges and barriers, spanning various domains, including familial, medical, socioeconomic, educational, and legal. In Brazil, these challenges are often compounded by socio-economic disparities and healthcare system limitations.

Family dynamics play a crucial role in post-hospital discharge home care. While some families possess strong support systems and caregiving resources, others may lack the necessary knowledge and skills to provide adequate care, leading to increased burden and potential gaps in patient management. Additionally, familial conflicts, financial constraints, and cultural beliefs can further hinder the implementation of effective home care plans. Medical factors, such as the severity and complexity of the patient's condition, significantly impact the feasibility and success of home care. Patients with chronic illnesses or debilitating conditions may require specialized equipment, continuous monitoring, and skilled nursing care, which may not always be readily available in the home setting. Moreover, comorbidities and disease progression can exacerbate functional limitations, necessitating tailored interventions and multidisciplinary collaboration. Socioeconomic factors play a pivotal role in shaping the accessibility and quality of home care services. In Brazil, income, education, and healthcare infrastructure disparities contribute to unequal access to medical resources and supportive services. Limited financial resources may impede the acquisition of essential medical supplies and medications, while inadequate housing conditions and sanitation facilities can compromise patient safety and well-being. Educational barriers further exacerbate the complexities of home care, particularly among populations with low health literacy and limited access to healthcare information. Misunderstandings regarding medication administration, dietary restrictions, and symptom management can lead to suboptimal treatment outcomes and increased risk of complications. Addressing these educational gaps requires targeted interventions, including patient education programs and health literacy initiatives. From a legal perspective, navigating the regulatory framework surrounding home care services presents additional challenges. In Brazil, legal ambiguities and bureaucratic hurdles may impede the timely provision of home care, delaying patient discharge and exacerbating healthcare costs. Furthermore, legal constraints regarding caregiver responsibilities, consent procedures, and liability issues necessitate clear communication and collaboration between healthcare providers, patients, and their families.

## FINAL CONSIDERATIONS

The relationship between post-ICU disabilities and adherence to clinic-based follow-up care remains unclear. Nevertheless, it seems that the burden of disabilities resulting from critical illness may prevent many patients from attending scheduled follow-up appointments at clinics.^(36)^ The traditional model of post-ICU care, which largely relies on clinic visits, does not appear to meet the needs of a substantial portion of patients who could benefit more from these services. As a result, alternative care models, such as home health care, telemedicine, and remote telemonitoring, may offer valuable solutions to address the needs of this patient population and help reduce health disparities (Table 3).

**Table 3:** Characteristics of patients for each post-hospital discharge and care alternative

Discharge to	Patient characteristics
Ambulatory clinics	Stable condition: patients with a stable medical condition do not require continuous intensive care Mobility: patients who can move independently or with minimal assistance Self-care ability: patients capable of performing most personal care activities Family support: the presence of a family or social support network that can help if necessary Need for medical follow-up: patients who require periodic monitoring or follow-up appointments to adjust treatments
Home care	Moderate condition: patients who may have stable medical conditions but still need assistance with some daily activities Medical assistance needed: patients who require continuous medical care that can be provided at home, such as intravenous medication administration, physical therapy, etc. Ability to remain at home: patients who can benefit from a familiar and comfortable environment for recovery Presence of caregivers: professional or family caregivers are needed to be trained to provide care Need for remote monitoring: patients may be monitored through telemedicine
Post-acute care facilities	Severe but stable condition patients who have transitioned from a critical condition but still need specialized care before returning home or to a less intensive environment Intensive rehabilitation: patients needing intensive rehabilitation (physical and occupational therapy) to regain functionality Continuous medical assistance: patients who require ongoing monitoring and medical treatment that cannot be provided at home Transition period: patients who need a transitional phase between the hospital and final discharge to home or another long-term care facility
Nursing homes	Chronic or degenerative condition: patients with chronic or degenerative medical conditions requiring long-term care High dependency: patients with a high level of dependency, needing assistance with all daily activities (bathing, feeding, mobility) Continuous monitoring: need for continuous monitoring by nursing staff Lack of adequate family support: patients who do not have a family or social support network that can provide necessary care at home Need for specialized care: patients who require continuous specialized care that cannot be provided in a home environment
Telehealth (telemedicine)	Stable condition: patients with a stable medical condition do not require frequent physical examinations or procedures Tech-savvy or support available: patients who are comfortable with technology or have support from family members or caregivers to navigate telehealth platforms Mild symptoms: patients with mild symptoms that can be monitored and managed remotely without needing in-person visits Chronic disease management: patients with chronic conditions that require regular monitoring, medication adjustments, and follow-up, which can be effectively managed through telehealth Post-operative follow-up: patients recovering from surgery who need follow-up consultations that can be conducted via video call to assess healing and address concerns Mental health support: patients needing mental health services, such as therapy or counseling, which can be delivered through secure video conferencing platforms Geographical barriers: patients living in remote or underserved areas with limited access to healthcare facilities, making telehealth a convenient option for receiving care Immunocompromised patients: patients who need to minimize exposure to infections, such as immunocompromised individuals, for whom in-person visits might pose a higher risk

Providing post-discharge home care involves navigating a range of complex factors, including familial, medical, socioeconomic, educational, and legal considerations. Overcoming these barriers requires a holistic approach integrating thorough assessments, personalized care planning, active community engagement, and advocacy for policies promoting equitable access to high-quality home care services for all patients.
